# The Digital Evolution of the Medical Black Bag: Environmental Scan With Trend Analysis and Horizon Scanning

**DOI:** 10.2196/93861

**Published:** 2026-08-06

**Authors:** Gellért Katonai, Nora Arvai, Bertalan Mesko

**Affiliations:** 1 Kálmán Laki Doctoral School of Biomedical and Clinical Sciences, University of Debrecen, Egyetem tér 1, Főépület földszint 15/A, Debrecen, 4032, Hungary, 36 52-258-010 ext 58010; 2 Department of Family Medicine, Semmelweis University, Budapest, Hungary; 3 The Medical Futurist Institute, Budapest, Hungary; 4 Meducation Hungary Kft, Budapest, Hungary

**Keywords:** primary care, general practice, point-of-care diagnostics, portable diagnostic devices, digital health, medical black bag, horizon scanning, trend analysis, artificial intelligence, connected devices

## Abstract

**Background:**

The medical black bag is synonymous with physicians, especially general practitioners, who are expected to be ready to provide care across settings. The content of the devices they use will likely expand due to the proliferation of digital tools. As portable diagnostics diversify, guidance is increasingly needed on which tools clinicians should choose and what this shift may mean for the physical examination and point-of-care assessment.

**Objective:**

This study aimed to map the current, the possible, and the future content of the medical black bag using anticipatory methods, and to provide a general, practice-oriented outline of how portable diagnostic technologies may evolve in primary care.

**Methods:**

National equipment lists and the World Health Organization’s MeDevIS database were compiled and filtered to define a contemporary reference set of reusable portable diagnostic instruments relevant to generalist practice. A 1-year trend analysis using major professional and medical technology news sources was conducted to identify possible additions, screening for devices with diagnostic relevance, portability, digital capability, market presence, and evidence visibility. To extend the outlook to the next decade, we performed a horizon-scanning exercise using the same review period. These devices were grouped into thematic categories.

**Results:**

National equipment recommendations and World Health Organization lists yielded a stable core set of diagnostic tools used in routine primary care practice. Trend analysis and horizon scanning expanded this set by identifying possible and future additions of portable medical devices that can be used at the point of care. Overall, the identified technologies were increasingly digital, diverse, connected, and in some cases, AI-supported, reflecting a trajectory toward more integrated and data-enabled diagnostics.

**Conclusions:**

The medical black bag is likely to evolve from a stable set of familiar instruments toward a broader toolbox of portable and connected diagnostic devices. While these tools may expand the scope of bedside assessment and enable more reproducible and shareable clinical signs, their value depends on appropriate validation, usability, workflow integration, training, and supportive financial and organizational conditions. Regular evidence-informed updates of equipment recommendations, alongside practical implementation support, may help primary care systems adopt useful innovations while preserving the human dimensions of clinical care.

## Introduction

The physician’s black bag is a compact kit designed to hold the tools and medicines needed for treatment at the point of care. Tracing back to early medical practice, its concept became firmly rooted in the physicians’ in-house visits carrying essential medicines and basic diagnostic devices. While current emergency medical services deploy specialized packs for life-threatening situations, the black bag remained synonymous with the general practitioner, not only as a toolset but also a cultural emblem of medical identity that signifies continuity of medical presence across diverse settings and geographical locations. At the same time, the fast pace of technological and organizational change is redefining our understanding and our models of care. Using an established concept, the physician’s black bag as a conceptual reference point allows us to trace how medical tools adapt to shifting contexts [1,2].

Early in the twentieth century, the bag contained the essential tools for bedside examination, many of which remain in use today. Some instruments have changed in form but not in purpose, as seen in the replacement of mercury sphygmomanometers and thermometers with digital versions from the 2000 s onward. These gradual substitutions reflect refinement more than reinvention, showing how diagnostic practice adapts while preserving continuity. New devices, such as the finger pulse oximeter, have become popular both in primary care and even home settings, particularly during the COVID-19 pandemic. These historical examples provide a rationale for exploring how the next generation of portable instruments may further extend and reshape the physician’s diagnostic toolkit [2-6].

To map the current landscape, our policy review shows that no international standard defines what the physician’s black bag should contain, and in many health systems, no formal reference exists at all. In some countries, such as Australia and New Zealand, professional organizations outline the expected diagnostic equipment [7,8]. In other places, lists function primarily as professional guidance rather than legal requirements. Physicians therefore decide individually which instruments to include, based on their clinical experience, local context, available technology, and financial considerations. As portable devices and their diagnostic capabilities proliferate, the rigorous evaluation of their clinical relevance and practical feasibility becomes essential to ensure that decisions are based on evidence rather than marketing dynamics alone, especially when formal guidance on their usefulness is mostly unspecified.

To address the uncertainty arising from the evolving adaptation of professional guidelines in the context of the rapid digital health revolution, a forward-looking approach is required. Futures methods, such as trend analysis and horizon scanning, provide structured means to identify emerging technologies and anticipate their potential value in health care [9,10]. While these approaches are well established in policy and innovation research, they remain underused in medicine. Applying them to map and discover portable diagnostic instruments makes it possible not only to describe current practice but also to anticipate how new devices may shape the future of point-of-care assessment, and support decision-makers when updating professional equipment recommendations [11].

In this study, we analyze the contents and potential future of the physician’s black bag by examining portable diagnostic devices from three perspectives: tools already in common use, technologies and devices that could already be included in the bag, and early innovations that may become feasible by the next decade. Revisiting the black bag through this framework highlights broader changes in clinical reasoning, point-of-care diagnostics, and the organization of future health care delivery [5].

## Methods

### Overview

Due to the nonstandardized content of the medical black bag, we first aimed to establish a general overview of the diagnostic devices that are widely recommended for diagnostic use to physicians globally. To do this, we used the World Health Organization’s Priority Medical Devices Information System (MeDevIS, version: 2025 v2.0) to identify relevant instruments. MeDevIS is an open-access, web-based tool developed to consolidate evidence-based lists of essential medical devices for disease prevention, diagnosis, and treatment across various levels of care. The database integrates technical specifications from multiple World Health Organization publications and allows users to filter devices by intended use, health intervention, care setting, and target population. Its detailed filtering capabilities and scope make it well-suited to identifying core diagnostic equipment.

To identify reusable portable diagnostic tools relevant to generalist practice, we applied filters within MeDevIS to identify devices that fit these criteria (described in Table 1). The resulting list was then compared with mandatory or recommended equipment lists from several countries to ensure contextual validity, producing a consolidated set of devices for further analysis. The complete filtered list is provided in Multimedia Appendix 1.

**Table 1.** MeDevIS filtering criteria. Filtering criteria applied in the World Health Organization’s Priority Medical Devices Information System (MeDevIS) to identify reusable, portable diagnostic devices relevant to general practice (primary care outpatient and outreach settings).

Filter category	Selected value
Primary use	Diagnosis or measurement or monitoring
Intended population age	All ages, adults (20‐64 y), later adults (over 64 y)
Intended population sex	All
Service delivery platforms or health care levels	General outpatient (health post, health center) and outreach services for primary care
Health care unit	Outpatient care, prehospital care
Type of medical device or related product	Medical equipment
Capital equipment	Yes
Reusable	Yes
Knowledge level	Basic, general clinical

After defining the reference set of essential diagnostic instruments through the MeDevIS database, the next phase involved environmental scanning to identify signals that could inform future additions to this set. In this study, environmental scanning refers to a structured review of external information sources to detect relevant developments, innovations, and contextual changes in diagnostic technologies. For the environmental scan, we purposively selected major professional and industry news sources with consistent publication frequency, a clear focus on medical devices and digital health, and publicly accessible reporting that supports traceable identification of diagnostic technology developments. The included outlets were MobiHealthNews, MedTech Dive, MassDevice, Med-Tech Insights, BioWorld, Medical Device Network, Digital Health, and Fierce Medtech [12-19]. These outlets were reviewed chronologically by the authors over a period spanning more than 1 year.

These outlets were selected because they provide consistently curated, publicly accessible reporting on medical device developments and pathways toward use in clinical practice. This made them suitable for systematically identifying signals relevant to point-of-care diagnostics.

Within this environmental scan, trend analysis was used to identify and describe recurring developments in news coverage of portable, digitally enabled diagnostic technologies. News items were screened against the inclusion criteria in Table 2.

**Table 2.** Medical device news filtering criteria for emerging tool identification. Inclusion criteria used to screen medical technology news items in a one-year trend analysis to identify portable, digitally enabled diagnostic devices relevant to point-of-care use in primary care.

Criterion	Description	Rationale
Diagnostic relevance	The device must serve a clear diagnostic or monitoring purpose rather than consumer wellness use.	Ensures inclusion aligns with the black bag’s diagnostic focus.
Portability	The device must be handheld or easily transportable without the need for fixed installation.	Reflects the physical constraints of a physician’s bag.
Digital capability	The device must feature digital measurement, signal processing, or data connectivity (eg, app link and wireless transfer).	Captures the shift toward connected, data-enabled diagnostics.
Availability or market presence	The device must have reached commercial availability in at least 1 country.	Ensures that the inclusion is feasible.
Evidence visibility	The device must have been covered by credible medical technology news outlets or peer-reviewed reports within the defined time window.	Ensures inclusion based on verifiable, traceable sources.

Items meeting these criteria were discussed in a consensus-based coauthor workshop where we presented each other the included pieces of news, resolved borderline cases, and confirmed the final set.

After the final set of items was agreed, we used the included articles to abstract a consolidated list of the devices and device types described in the reporting, and grouped them based on shared functional characteristics. These groupings formed the basis of the technology directions described in the trend analysis and the near-term opportunities for updating the physician’s black bag. The selected sources and the final list of devices with short descriptions and their evidence levels are provided in Multimedia Appendices 2 and 3.

Using the same environmental scan as an input stream, the horizon scanning exercise focused on longer-term possibilities by identifying early-stage or weak-signal developments that could plausibly shape the medical black bag by the next decade. This method complemented the trend analysis by capturing early-stage innovations that are not yet clinically validated or commercially available but show potential for diagnostic application at the point of care.

Reports were drawn from the same news sources over the review period. Candidate early signals were screened using the inclusion criteria in Table 3 and discussed within the coauthor team to resolve borderline cases and confirm the final set.

**Table 3.** Medical device news filtering criteria for horizon scanning. Inclusion criteria used to identify early-stage, noncommercially available weak signals that have potential diagnostic application in PHC settings.

Criterion	Description	Rationale
Diagnostic relevance	The technology must demonstrate potential to support diagnostic or monitoring functions rather than wellness or consumer use.	Ensures inclusion aligns with the diagnostic focus of the physician’s black bag, even for early-stage concepts.
Portability	The device must be handheld or easily transportable without the need for fixed installation.	Reflects the spatial and practical constraints of point-of-care diagnostics and the portable nature of the bag.
Digital capability	The innovation must rely on digital sensing, signal processing, or algorithmic interpretation as a core feature.	Captures the ongoing shift toward data-driven and AI-enabled diagnostic modalities.
Developmental stage or emergence	The technology must be in prototype, pilot, or early regulatory-pipeline phase, indicating potential but not yet established availability.	Identifies weak signals representing innovations that may mature into future diagnostic tools.
Evidence visibility	The signal must have been reported in credible medical technology news, early research communications, or regulatory previews within the defined time window.	Ensures that the identified signals are traceable, verifiable, and grounded in publicly accessible information.

The resulting set of early signals was reviewed collaboratively to consolidate overlapping developments and group related innovations into overarching thematic categories. This process resulted in a structured outlook on diagnostic technology modalities that may transition from prototype to practical implementation over the next decade. The detailed list of identified signals, with corresponding sources and short descriptions, is available in Multimedia Appendix 4.

### Ethical Considerations

Ethics approval was not required because this study analyzed publicly available information and did not involve human participants, patient-level data, or identifiable personal information.

## Results

### Overview

The results of our study are structured around 3 distinct groups of diagnostic instruments associated with the physician’s black bag. Combined, these inventories suggest how the devices in the bag may diversify and evolve, reflecting both continuity and new diagnostic opportunities. First, we documented the contemporary set of 11 devices through a literature review based on internationally accepted lists of essential diagnostic equipment.

**Figure 1.**
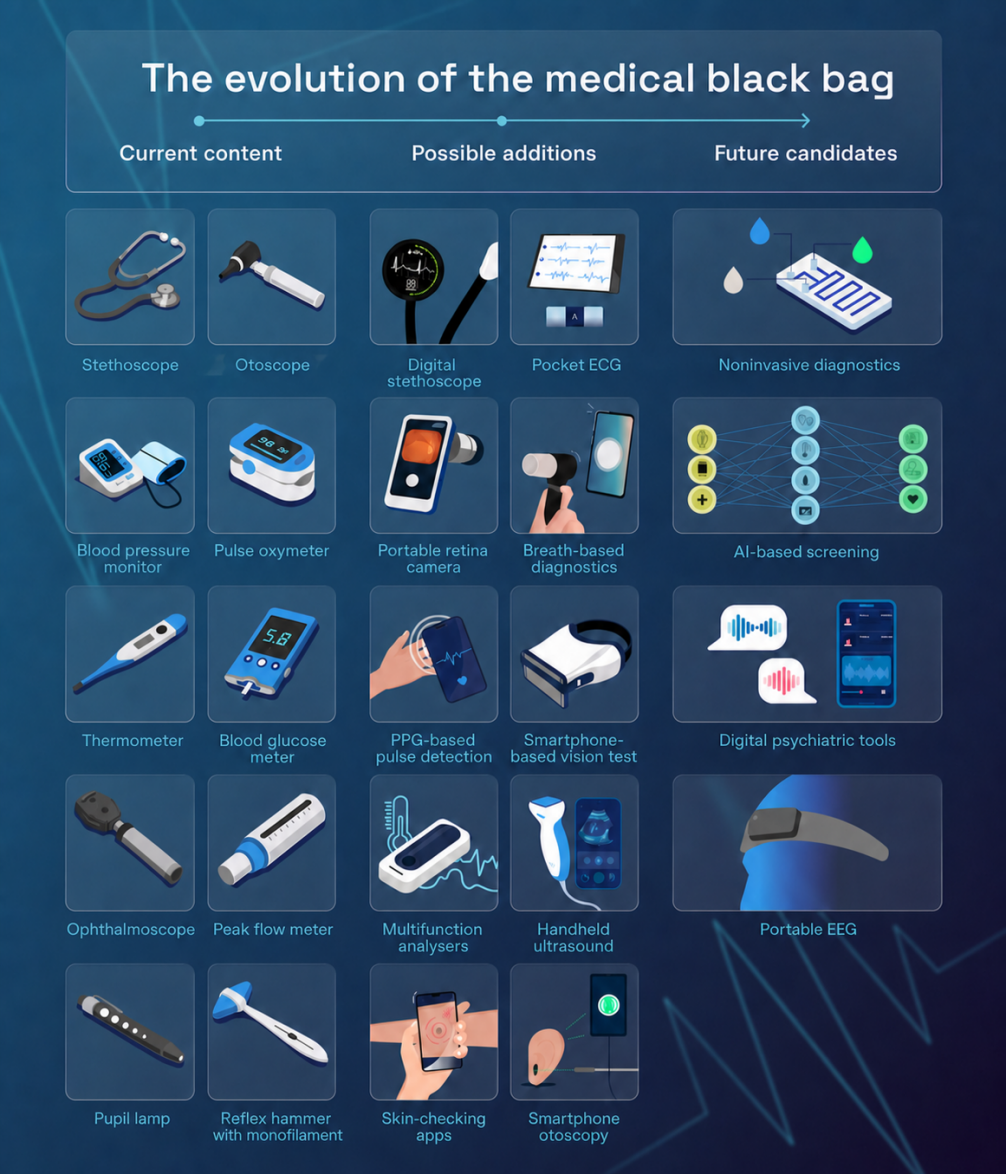
Visual overview of diagnostic instruments associated with the physician’s black bag, grouped as current contents (derived from WHO MeDevIS filtering and national equipment lists), near-term possible additions (identified through the trend analysis of medical technology news sources), and future candidates (identified through horizon scanning with an outlook up to the next decade). ECG: electrocardiogram; EEG: electroencephalogram; PPG: photoplethysmogram.

**Figure 2.**
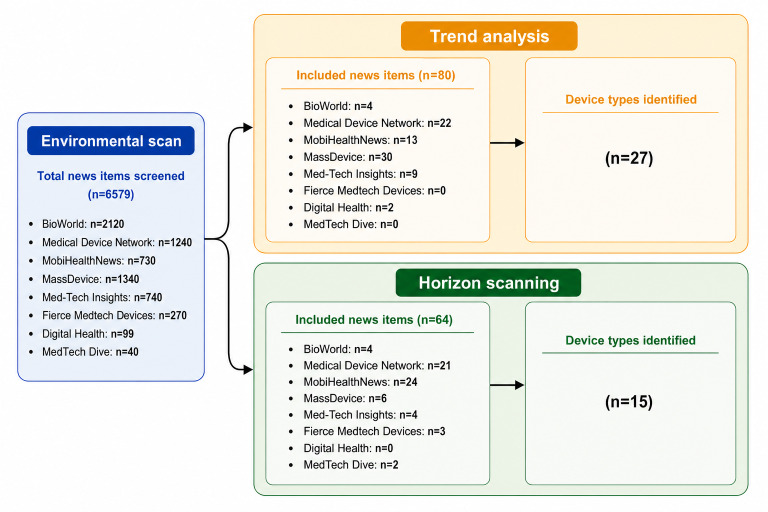
Flow diagram of the news-based environmental scan and downstream analysis. News items published within the study time window were screened across predefined outlets, and counts screened per outlet are reported. Two analytic subsets were then derived from the screened corpus: trend analysis (included news items describing market-available portable diagnostic devices) and horizon scanning (included news items describing emerging or early-stage diagnostic technologies). For each subset, the figure reports the number of included news items by outlet and the subsequent abstraction outputs (device types and themes grouped from the included items).

Second, we conducted an environmental scan of 6579 news items published during the review period across the included outlets (BioWorld n=2120, Medical Device Network n=1240, MassDevice n=1340, Med-Tech Insights n=740, MobiHealthNews n=730, Fierce Medtech Devices n=270, Digital Health n=99, MedTech Dive n=40). From the environmental scan, 80 news items met the inclusion criteria for trend analysis. From these, we consolidated 27 device types and grouped those into 4 functional groups. The full device list is provided in Multimedia Appendix 3, and their visual representations are shown in Figure 1. Figure 2 demonstrates the flow diagram of this process.

Third, to extend the future outlook to the next decade, we applied horizon scanning to identify weak signals and early-stage innovations. From the environmental scan, 64 news items met the inclusion criteria for horizon scanning and were retained. These were consolidated into 15 emerging technology themes, which were further grouped based on shared characteristics. The full list of identified signals and consolidated themes is provided in Multimedia Appendix 4. The flow chart of this process is demonstrated on Figure 2.

### Current Contents of the Black Bag

Based on our synthesis of equipment lists, we identified a core group of instruments recurring within the black bag. It includes the stethoscope, blood pressure monitor, and thermometer for measuring vital signs. Examination of the eyes, ears, and mucosal surfaces relies on a set of visualization instruments, including the ophthalmoscope, otoscope, and a portable light source, usually a penlight. Additional point-of-care physiological testing can be performed with the pulse oximeter (to measure oxygen saturation and pulse rate), blood glucose meter (to assess glycemic control), and the peak flow meter (to evaluate peak expiratory flow). Basic neurological examination relies on the reflex hammer with monofilament and tuning fork. Collectively, these devices represent a set of instruments that are familiar to physicians in everyday clinical practice.

### Possible Additions to the Black Bag

#### Overview

Our trend analysis yielded a variety of commercially available devices that are not yet included in equipment lists but could accompany (or, in some cases, replace) the traditional arsenal of the black bag. Due to the diverse nature of the devices identified, we present them in 4 categories: enhanced versions of familiar instruments, portable clinical equipment, smartphone-based diagnostic tools, and multifunctional analyzers.

#### Enhanced Versions of Familiar Instruments

Digital stethoscopes extend traditional auscultation by enabling both the recording and subsequent analysis of heart and lung sounds. They provide amplification, reduction of background noise, and often visualization of the waveforms on the chassis or on the display of connected hardware. These devices can be linked to smartphones or tablets, enabling recordings to be stored or shared as part of the patient record. Many models also allow automated arrhythmia detection and, in some cases, include a single-lead electrocardiography (ECG) channel, expanding their role from listening to combined cardiopulmonary screening.

Oscillometric blood pressure monitors are widely used for blood pressure assessment. Their connected versions (cellular or Bluetooth) not only transmit data to smartphones or health platforms and track long-term trends, but may also integrate single- or few-lead ECG capabilities that can automatically detect irregular rhythms and other abnormalities, thereby expanding their diagnostic use.

Pulse oximeters represent another cornerstone of vital-sign monitoring. Recent developments include the use of multiple wavelengths and the incorporation of laser-based emitters designed to support consistent readings in the presence of motion and low perfusion, across diverse skin tones. These efforts have been accelerated by increased regulatory scrutiny, prompted by reported issues in reading accuracy. In parallel, cellular connectivity and data sharing further enhance the accessibility and clinical use of the measurements provided.

Digital peak flow meters, glucose meters, and thermometers follow a similar trajectory. While their traditional forms required patients to manually record readings, connected versions now automatically capture results, sync with smartphones or cloud platforms to make measurement data available to clinicians in real time.

#### Portable Versions of Clinical Equipment

Alongside improvements to existing devices, a variety of new portable diagnostic solutions have entered the market. Some of these take diagnostic modalities found in hospitals and place them into small, easy-to-carry units, while others apply vastly different approaches to diagnosis.

Electrocardiography is listed as a basic requirement for most clinical practices, but conventional devices are bulky and mostly stationary. To bridge this gap, pocket-sized, 12-lead systems have become commercially available. Most of them use streamlined configurations (either single-cable connections or preconfigured patches) to produce standard tracings. Many devices connect directly to smartphones or tablets, which can act as the display and the storage platform, and allow results to be transmitted or linked to patient records. Automated rhythm interpretation is typically included; in some cases, AI-based algorithms have been validated against clinician performance for detecting common abnormalities. In addition to full 12-lead ECG systems, single-lead or partial-lead devices have been available for rapid screening for rhythm disturbances.

Handheld ultrasound machines, paired with a smart device, allow visualization of internal structures at the point of care, enhancing point-of-care diagnostic capabilities. Although these devices have been available for years, advances in image resolution and steadily declining costs have increased their availability. Many now include software with regulatory-cleared AI features such as probe-positioning assistance, anatomical labeling, and automated measurement, potentially reducing training time for physicians. Images taken with the device can be stored locally or in compatible systems and shared for further evaluation. In parallel, specialized ultrasound patches have been introduced for measuring carotid artery flow, providing additional information on arterial status.

Traditionally, lung function testing relies on large equipment, but newer modalities have been adapted into handheld devices. Portable spirometry systems offer validated measurements of airflow (and, in some systems, lung volumes) in small, connected formats, bringing spirometry closer to point-of-care use in generalist settings. Many devices add smartphone and cloud connectivity and algorithmic support for longitudinal monitoring and integration with digital health records. Other approaches analyze tidal CO₂ waveforms during breathing and, in some cases, use AI to support rapid chronic obstructive pulmonary disease (COPD) assessment.

Portable, fractional exhaled nitric oxide (FeNO) analyzers support asthma diagnosis and management by providing a noninvasive biomarker of airway inflammation. This principle illustrates how exhaled breath can serve as a readily accessible source of diagnostic information. Similar approaches include sensors for exhaled carbon monoxide (CO), used in smoking cessation programs or to assess exposure, hydrogen (H₂) breath tests for gastrointestinal disorders such as lactose intolerance or small intestinal bacterial overgrowth, and breath alcohol tests for alcohol intoxication monitoring.

Cardiotocography is another diagnostic modality that was previously tied to maternity units in hospitals. Portable devices that combine fetal heart rate and uterine contraction monitoring can be small enough to fit in a physician’s bag and offer the potential for point-of-care antenatal assessment. Handheld retinal cameras bring microvascular screening closer to community and home settings without the need for immediate referral to ophthalmology, with some models offering semi-automated image grading at the point of care.

#### Smartphone-Based Diagnostic Tools

Smartphones have become versatile diagnostic platforms, capable of measuring parameters either via built-in sensors or through attachments paired with compatible software, which in some cases can function as regulated medical devices. Their portability and cloud connectivity allow them to serve as a hub for collecting, processing, and sharing patient data at the point of care.

Modern mobile devices’ high-resolution cameras and expanded storage support the visualization and recording of clinically useful images, including smartphone-based ophthalmoscopy and otoscopy. Procedures that were previously dependent solely on lenses, the physician’s technique, and judgment can now be augmented with digital images. Some devices align the phone with a bracket and traditional lenses; other otoscopes use a miniaturized camera with a flexible tip connected to the phone via a cable or wirelessly.

In addition to visualization, some smartphone apps have received regulatory clearance for physiological measurements using the phone’s camera. These include estimating blood pressure from optical signals, measuring pulse and respiratory rates, and, in some cases, detecting rhythm irregularities without external devices. Cough-sound analysis using the onboard microphone represents a newer approach for differentiating respiratory conditions.

Screening for common conditions, such as vision disturbances and skin cancer, has also been adapted for smart devices. Available attachments or stand-alone apps can estimate refractive error, test visual acuity, or monitor central vision changes in retinal disease. Similarly, skin-checking apps and attachment tools capture images of lesions and analyze them using AI-assisted software to support cancer risk assessment. Some of these applications have been incorporated into clinical pathways in specific settings.

#### Multifunction Analyzers

Handheld devices that can perform multiple types of measurements are also on the rise, with the ability to combine several individual instruments into one. Common combinations include cardiorespiratory measurements (few-lead ECG, pulse oximetry, and respiratory rate), vital signs (temperature and blood pressure), and digital auscultation. Some devices further extend into specialties such as otoscopy or dermatoscopy, providing an integrated solution with centrally stored records. These systems are typically physician-operated and applied directly to the relevant body site, allowing rapid spot assessments during acute care encounters.

Many of these parameters can also be measured with small, dedicated wrist- or patch-style devices. These discrete systems can provide real-time (or continuous) recording of heart rate, respiratory rate, oxygen saturation, skin temperature, and blood pressure. Unlike handheld tools, they are applied once and left in place, offering immediate multiparameter readings with minimal setup, which can support short-term monitoring in acute settings.

### Future Candidates

As we look ahead to the next decade, several diagnostic approaches that are currently experimental or in early development may become mature and portable enough to be included in the physician’s bag, representing new ways of measuring or analyzing health data. Compared with the wide range of commercially available tools identified through trend analysis, the horizon scanning exercise yielded a smaller set of early-stage innovations. This is expected because only about 45% of new medical devices described in early clinical studies ultimately receive regulatory clearance or approval, so many early signals will not translate into market-ready tools [20].

One area of development is noninvasive diagnostic testing, including both host biomarkers and direct pathogen detection. There are efforts underway to develop and evaluate saliva-based sensors as a practical way of measuring biochemical information during point-of-care assessments. Breath analysis is also advancing; prototypes of “volatile biopsy” devices aim to detect conditions such as tuberculosis through exhaled biomarkers. Compact multiplex point-of-care platforms are being developed for at-home testing of multiple pathogens in a single device. CRISPR-based diagnostics align with this direction, leveraging similar sampling approaches to enable highly specific nucleic acid detection with portable readouts (eg, lateral-flow strips or smartphone-based readers). Together, these approaches suggest that biochemical diagnostics may increasingly be delivered in rapid, portable formats without the need to draw blood.

AI-based screening and diagnostic methods have the potential to change the black bag substantially by the next decade. Conventional retinal imaging systems are increasingly embedding machine learning algorithms to support screening for eye disease and to derive additional biomarkers from image patterns. ECG-specific algorithms have also been developed to detect certain systemic conditions beyond arrhythmias, such as electrolyte disturbances or fluid imbalance. AI-enabled spectroscopy devices for melanoma screening that are already used in hospital settings are becoming increasingly portable and may eventually be feasible for inclusion in the black bag. Locally run language models, trained on health care-domain knowledge and integrated with sensor data, could provide rapid decision support in settings without reliable internet access.

Psychiatric evaluation may also be affected by the next wave of innovation. AI models have shown the ability to detect signals associated with depression in voice recordings, suggesting new approaches to supporting assessment of affective disorders. Analogous to computer-based tests, emerging digital psychiatric tools using eye tracking, speech, or interaction patterns could support triage and differential assessment of acute presentations outside specialized settings. While not replacing comprehensive psychiatric evaluation, such tools could provide structured input to guide referral or initial management, complementing self-reported questionnaires.

Smartphone-based tools also appear to be expanding further, with multiple efforts underway to broaden diagnostic applications, including specialized attachments to detect UV-related eye damage, applications that support stroke assessment, and innovations that transform the phone itself into a stethoscope or an audiometry tool.

Efforts to downsize traditionally clinic-based diagnostic modalities are evident across several domains, reflecting a trend toward more portable and accessible tools. Portable EEG devices have been developed that use a limited number of leads to monitor brain activity in clinically relevant contexts, and several incorporate AI-based algorithms for seizure detection and automated interpretation. Smartphone- and sensor-based tools are also being applied to the assessment of gait and mobility, providing quantitative measures of joint mobility and asymmetry, including differences in limb size.

## Discussion

### Principal Findings

Our analysis categorized diagnostic instruments associated with the physician’s black bag into 3 groups, reflecting current contents, near-term possible additions, and longer-term emerging technologies. The results revealed a stable core of portable diagnostic tools that continues to define physicians’ daily practice, alongside an expanding range of devices that broadens the scope of point-of-care assessment. The evolution of these devices points toward increasingly digitalized, integrated, connected, and AI-supported diagnostics and screening that expand what is feasible at the point of care. These findings highlight an ongoing diversification of diagnostic tools, driven by advances in compactification, connectivity, and technological efficiency. At the same time, this evolution raises important uncertainties regarding validation, usability, and integration into primary care workflows.

### Rethinking the Physical Examination

The traditional physical examination relies on the physician’s sensory perception: inspection, palpation, percussion, and auscultation, each supported by well-established analog tools. Building on these traditional skills, the emerging generation of diagnostic instruments can enhance these modalities, allowing health care professionals to perceive and interpret clinical signs that were previously hard to detect reliably. Digitized clinical signs may be amplified, visualized, and quantified, translating perception into measurable and interpretable data.

This digital augmentation can introduce a new level of reproducibility and traceability, supporting the accuracy and reliability of diagnostic processes. Findings that were once fleeting and based on personal interpretation can increasingly be recorded, stored, and compared across clinical encounters, or shared for further analysis. Repeated measurements may accumulate into patient-specific diagnostic stories, turning isolated encounters into a more quantified and longitudinal observation process from the outset.

In addition to digital augmentation, access to novel physiological and biochemical biomarkers previously unavailable at the bedside can expand the possibilities of acute assessment. Miniaturized sensors and portable analytical platforms may enable clinicians to measure parameters that once required hospital infrastructure or specialized training, within minutes. This more immediate feedback can narrow the gap between detection and decision, allowing earlier and more confident triage in community and home settings.

**Figure 3.**
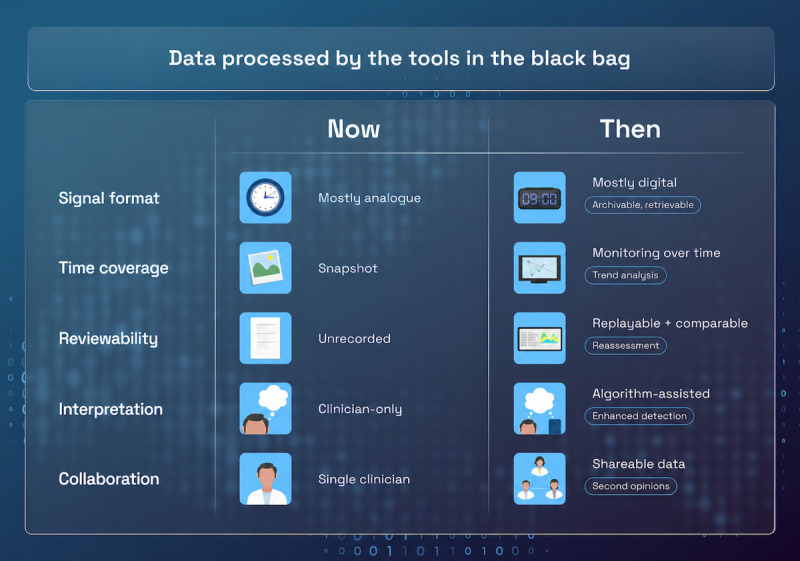
Rethinking the physical examination: conceptual summary of how digitized clinical signs can be amplified, quantified, recorded, and shared, enabling reproducible and longitudinal point-of-care assessment and, in some cases, AI-assisted interpretation in primary care.

These processes can be further supported by AI-assisted interpretations that highlight features not easily perceived in routine examination. Such outputs can provide additional cues that clinicians may integrate into their immediate assessment. These shifts in the diagnostic act are summarized in Figure 3.

### Implications and Pitfalls for Practice

Digital diagnostic devices can influence the initial clinical impression and the subsequent course of decision-making by providing additional, decision-relevant information during the consultation. Evidence from handheld ultrasound illustrates this potential: point-of-care imaging has been shown to alter management and increase diagnostic confidence in a meaningful proportion of primary care consultations where it was used [21,22]. Digital diagnostic devices can influence the initial clinical impression and the subsequent course of decision-making by providing additional, decision-relevant information during the consultation. Evidence from handheld ultrasound illustrates this potential: point-of-care imaging has been shown to alter management and increase diagnostic confidence in a meaningful proportion of primary care consultations where it was used [23,24].

The ability to capture multiple data-rich signals in a single step has the potential to make examinations more efficient. Stethoscopes with integrated ECG leads exemplify this possibility: by combining auscultation and electrophysiological recording, they can detect cardiac abnormalities without requiring separate procedures or additional equipment [25,26]. Yet integrating such tools into practice presents challenges. Clinicians frequently experience workflow disruption and increased cognitive burden when new digital tools alter familiar routines, and systematic reviews show that the early phase of implementing smart health care technologies is often associated with reduced productivity and heightened stress before any efficiency gains emerge. These transitional difficulties may need careful consideration when these tools are being deployed in real-world settings [27,28].

With the use of these devices, referral pathways may also shift, as certain assessments become feasible within primary care rather than requiring specialist evaluation. Autonomous diabetic retinopathy screening illustrates this possibility: these systems have demonstrated the capacity to reliably identify referable disease at the point of care, enabling earlier decisions and reducing the need for routine specialist screening appointments [29,30]. However, such benefits only hold if the economic and organizational conditions support adoption. Evidence shows that substantial upfront costs, uncertainties around reimbursement, and concerns about added workload remain significant barriers for primary care clinics considering new digital systems. These constraints highlight that even when a diagnostic task can be performed autonomously within primary care, integration depends on financial viability and alignment with existing capacity and workflows [31]

Digitized diagnostic data can strengthen continuity and support collaboration with specialists by making clinical findings easier to store, revisit, and share when needed. Tele-ECG studies illustrate this potential, showing that records captured in primary care and transmitted to cardiology services can inform clearer decision-making and more coordinated follow-up [32,33]. The success of such information exchange depends on the reliability, interoperability, and security of the systems handling this data. Research shows that inconsistent data transfer between settings can interrupt clinical workflows, and recent analyses of health care cyber incidents highlight the vulnerability of digitized diagnostic information to security breaches [34,35].

These tools that generate objective, quantifiable signals can support more structured longitudinal monitoring in primary care. Portable spirometry has demonstrated that repeated measurements collected through compact devices can reliably track respiratory trajectories and guide treatment adjustments over time [36,37]. However, the usefulness of such monitoring depends on clinicians being sufficiently trained to acquire and interpret digital readings with confidence. Studies examining the uptake of point-of-care ultrasound in primary care show that many clinicians identify limited training time and uncertainty about maintaining competency as key obstacles to adoption. Similar concerns have been reported in atrial fibrillation screening initiatives, where primary care clinicians expressed hesitation about using sensor-based tools without clearer interpretive guidance and opportunities for supervised learning [38,39].

Providing visual output through these tools can help patients better understand what the clinician observes. Evidence from video-otoscopy demonstrates that showing recorded or magnified ear images during the consultation improves patient comprehension and satisfaction [40,41]. Yet the availability of such benefits is uneven across primary care. Adoption of digital diagnostic devices depends heavily on local resources, including access to equipment, stable connectivity, and the infrastructure needed to store and transfer device-generated data. These differences create variability in who can benefit from digitally enhanced explanations, leading to inconsistent patient experiences across practices [42,43].

### Current Status and Future Trajectories of the Identified Tools

Some digital diagnostics are already entering formal guidance and evaluation pathways within primary care. The most advanced examples include AI-assisted skin lesion assessment tools such as DERM, which has been conditionally recommended by the National Institute for Health and Care Excellence (NICE) for National Health Service (NHS) use pending further evidence, and SkinVision, evaluated in NICE Medtech Innovation Briefing (MIB311) and by the Dutch Healthcare Institute [44,45]. Biomarker-based innovations have also reached guideline integration: FeNO testing is now incorporated into both NICE NG245 and GINA asthma management recommendations [46,47]. Similarly, single-lead ECG devices like KardiaMobile are covered by NICE DG35 for detecting atrial fibrillation in primary care [48]. Beyond these examples, autonomous diabetic-retinopathy screening systems such as IDx-DR are included in the American Diabetes Association (ADA) Standards of Care [49]. Some NHS trusts have begun acquiring Butterfly iQ for point-of-care imaging, and the device is explicitly listed in NICE’s MedTech briefing as potentially usable in community and primary-care settings [50]. Emerging AI-assisted respiratory analysis, such as TidalSense, is currently undergoing NICE Early Value Assessment, signaling its potential for integration into community diagnostics [51]. Remote multisensor platforms like TytoCare, which have been piloted by multiple NHS trusts and regional networks, illustrate how connected diagnostic ecosystems are beginning to secure a place within public health services [52].

Among the tools already finding their way to formal primary-care pathways, several diagnostic modalities currently getting established in specialist settings appear close to possible adoption in general practice. AI-assisted electrocardiography provides one such example: systems such as PMcardio have demonstrated accuracy comparable to cardiologists for major ECG abnormalities and atrial fibrillation, with projects like the AMSTELHEART pilot illustrating how hospital-grade analysis could be integrated into community workflows [53]. Digital auscultation and phonocardiography with AI-based murmur detection are already used in cardiology programs to identify valvular disease and low ejection fraction, and recent trials show comparable diagnostic performance to expert clinicians, supporting potential translation into general practice [54]. Recent randomized evidence shows that an AI-assisted point-of-care cardiac ultrasound can support non-expert acquisition and interpretation of echocardiographic images, demonstrating high diagnostic accuracy [55]. AI-supported spirometry platforms, combining portable devices with cloud-based interpretation, have moved from respiratory clinics into validation in primary-care datasets, achieving strong sensitivity and specificity for COPD detection [56].

Further examples include connected video-otoscopy, increasingly used in ENT teleconsultation and remote-triage programs, showing that smartphone-linked otoscopy may support generalist or remote-care deployment. Digital tympanometry and audiometry systems are also shown to have promising results; hospital and telemedicine trials have demonstrated diagnostic performance comparable to conventional instruments, and smartphone-linked versions are under evaluation for broader screening and remote-care use [57,58].

### Policy Implications

The diversification of portable diagnostic devices calls for adjustments in how primary care systems select which devices to use. As shown in our findings, digital and connected tools expand the range of feasible examinations within routine consultations; therefore, equipment recommendations need regular updating to reflect both long-standing essentials and newer classes of portable technologies reaching clinical maturity. Clear guidance on which instruments are appropriate for routine use, which remain emerging, and how they should complement existing practice would help clinicians navigate this rapidly evolving landscape.

Broader adoption of these tools also depends on strengthening training and professional development. Many of the diagnostic modalities identified, including handheld imaging, digital auscultation, and sensor-based monitors, require familiarity with new forms of signal acquisition and interpretation. Structured educational frameworks and opportunities for skills maintenance will be essential to ensure that clinicians can use these technologies confidently and safely, without adding disproportionate workload in already resource-constrained settings.

Financial and organizational considerations remain equally important. Digital diagnostic devices often carry recurring costs related to software, consumables, or connectivity, making individual purchasing unrealistic for many practices. Shared-use arrangements, practice-network cooperation, or system-level support could help distribute costs and enable more equitable access across users. This integration also requires careful consideration of reimbursement models that recognize not only the initial purchase, but also the clinician time, maintenance, interpretation, and follow-up work involved in providing additional diagnostic services in routine care.

Several of the devices identified in this work are already beginning to appear in clinical pathways, highlighting the importance of systematic integration. As portable diagnostics become more capable, policy efforts should consider how they can be incorporated into referral processes, chronic disease management, and community diagnostic services. Clear liability frameworks are also needed to define professional responsibility when device-generated or AI-supported outputs influence referral, treatment, or reassurance decisions. Involving both clinicians and patients in these decisions will help ensure that new technologies support continuity, usability, and the core principles of primary care.

With the knowledge gathered from the deployment of recently adopted digital diagnostics, experience gained in clinical contexts can help create conditions that support the introduction of newer technologies still in experimental phases. As clinicians become familiar with digitally augmented examinations, connected data flows, and AI-supported interpretations, the organizational and technical conditions needed to evaluate further innovations become easier to establish. This accumulated familiarity may shorten the transition between early signals identified through horizon scanning and their eventual real-world assessment, as infrastructures for validation, training, and data handling are already partly in place. In this way, uptake of current digital tools not only changes present-day diagnostic practice but may also shape the readiness of primary care systems to integrate future point-of-care technologies as they mature.

Exposure and increased familiarity with these devices are not the only factors contributing to their success. Adoption is also shaped by decisions made across different levels of the health system, where actors often disagree on what they deem the central aims of care. Thus, for a technology to become routine depends on how clinicians, organizations, payers, regulators, and patients and care pathways align around questions of worth. The localization of treatments is also shifting as patients become the point of care, measurements become feasible at home, and hospital wards can go remote through monitoring and self-testing. These changes in organization also contribute to the emergence of new professional roles and the redistribution of diagnostic work.

Beyond these structural dynamics, these devices gain their “technology identity” through the lens through which they are interpreted, which shapes whether they are seen as legitimate clinical instruments, optional add-ons, or unnecessary gadgets, and, in that sense, the black bag remains more than a toolkit because its changing contents are socially legitimized as part of what counts as part of a future-ready practitioner [59-61].

### Limitations

This study has several limitations. The selection of devices and signals was based on publicly available sources, which may favor technologies with greater media visibility or commercial presence over equally relevant but less reported innovations. As horizon scanning and trend analysis inherently involve early and sometimes uncertain information, the inclusion of certain technologies reflects potential rather than established clinical readiness. The focus was restricted to diagnostic and monitoring instruments, excluding therapeutic and in vitro diagnostic devices, and the analysis was interpretive rather than evaluative. Consequently, the findings should be interpreted as indicative of emerging directions rather than exhaustive or prescriptive recommendations, and they should not be used as a stand-alone basis for decision-making.

### Future Directions

Further research is needed to understand how these technologies function in real-world general practice. Further studies should examine how digital devices influence consultation flow, diagnostic decision-making, follow-up patterns, and patient understanding, as well as their cost-effectiveness and implications for equity. Future qualitative interviews could also explore how clinicians and other stakeholders evaluate the feasibility and value of these emerging device categories in routine practice. Such evaluations would provide the evidence required to determine which tools meaningfully enhance care.

### Conclusions

The contents of the physician’s black bag have long symbolized the essential tools of clinical care. This study shows that while a stable core of diagnostic instruments persists, a growing number of digital and connected devices are expanding the scope of bedside assessment. Through a combination of literature review, trend analysis, and horizon scanning, we identified technologies that represent both continuity and transformation in diagnostic practice.

Together, these developments mark a gradual shift toward portable, multimodal, and algorithm-supported diagnostics that can accompany the physician across settings, from the clinic to the patient’s home. This evolution underscores the need for continuous, evidence-based reassessment of what constitutes essential diagnostic equipment, alongside the development of training and support structures that enable effective integration of new tools. The future of the physician’s black bag will depend not only on technological innovation but also on ensuring that these advances enhance rather than displace the human dimensions of primary care.

## Supplementary material

10.2196/93861 Multimedia Appendix 1 Medevis filtered devices with corresponding categories.

10.2196/93861 Multimedia Appendix 2 Signs that give the basis for future methods exploration separated by the trend analysis and the horizon scanning exercise.

10.2196/93861 Multimedia Appendix 3 The results of the trend analysis exercise.

10.2196/93861 Multimedia Appendix 4 The results of the horizon scanning exercise.

## Abbreviations

ADA: American Diabetes Association
ECG: electrocardiography
FeNO: fractional exhaled nitric oxide
MeDevIS: Priority Medical Devices Information System
NHS: National Health Service
NICE: National Institute for Health and Care Excellence
