# Supplementary material for: The Digital Evolution of the Medical Black Bag: Environmental Scan With Trend Analysis and Horizon Scanning
Source: J Med Internet Res. 2026 Aug 6;28:e93861. doi: 10.2196/93861 (PMC13446799; doi:10.2196/93861)
Supplement: Supplementary file 3 [file jmir-v28-e93861-s003.docx]

**Multimedia Appendix 3. Reports excluded**

| **Reference** | **Title and specific reason (amendments)** |
| --- | --- |
| **Wrong publication type (n = 2)** | |
| Absolom et al. (2017) | Electronic patient self-Reporting of Adverse-events: Patient Information and aDvice (eRAPID): a randomised controlled trial in systemic cancer treatment  doi: 10.1186/s12885-017-3303-8 |
| Gernier et al. (2023) | Impact of web application support versus standard management on adherence with adjuvant hormone therapy in patients treated for breast cancer: the WEBAPPAC study  doi: 10.1186/s12885-023-11242-1 |
| **Wrong design (n = 4)** | |
| Baik et al. (2020) | Patterns of Use of Smartphone-Based Interventions Among Latina Breast Cancer Survivors: Secondary Analysis of a Pilot Randomized Controlled Trial  doi: 10.2196/17538 |
| Masiero et al. (2024) | Support for Chronic Pain Management for Breast Cancer Survivors Through Novel Digital Health Ecosystems: Pilot Usability Study of the PainRELife Mobile App  doi: 10.2196/51021 |
| Poikonen-Saksela, et al. (2024) | Digital Self-Management Intervention Paths for Early Breast Cancer Patients: Results of a Pilot Study  doi: 10.1155/2024/8036696 |
| Tak et al. (2025) | Analysis of metabolic and quality-of-life factors in patients with cancer for a new approach to classifying walking habits: Secondary analysis of a randomized controlled trial  doi: 10.2196/52694 |
| **Wrong population (n = 17)** | |
| Absolom et al. (2021) | Phase III Randomized Controlled Trial of eRAPID: eHealth Intervention During Chemotherapy  doi: 10.1200/JCO.20.02015 |
| Akkol-Solakoglu et al. (2023) | Internet-delivered cognitive behavioural therapy for depression and anxiety in breast cancer survivors: Results from a randomised controlled trial  doi: 10.1002/pon.6097 |
| Bakitas et al. (2009) | Effects of a palliative care intervention on clinical outcomes in patients with advanced cancer: The Project ENABLE II randomized controlled trial  doi: 10.1001/jama.2009.1198 |
| Blair et al. (2021) | A Home-Based Mobile Health Intervention to Replace Sedentary Time With Light Physical Activity in Older Cancer Survivors: Randomized Controlled Pilot Trial  doi: 10.2196/18819 |
| Borosund et al. (2020) | Results from a randomized controlled trial testing StressProffen; an application-based stress-management intervention for cancer survivors  doi: 10.1002/cam4.3000 |
| Borosund et al. (2022) | Digital stress management in cancer: Testing StressProffen in a 12-month randomized controlled trial  doi: 10.1002/cncr.34046 |
| Cartwright et al. (2025) | Feasibility Randomised Controlled Trial of a New Digital Intervention (‘FRAME’) to Promote Resilience in Women Treated for Primary Breast Cancer  doi: 10.1002/pon.70217 |
| Fabi et al. (2022) | The immersive experience of virtual reality during chemotherapy in patients with early breast and ovarian cancers: The patient's dream study  doi: 10.3389/fonc.2022.960387 |
| Feufel et al. (2024) | iKNOW—Supporting the counseling of women with hereditary risk of breast and ovarian cancer with digital technologies: A randomized controlled trial  doi: 10.1016/j.gimo.2024.101892 |
| Hauffman et al. (2020) | Cocreated internet‐based stepped care for individuals with cancer and concurrent symptoms of anxiety and depression: Results from the U-CARE AdultCan randomized controlled trial  doi: 10.1002/pon.5489 |
| Holtdirk et al. (2021) | Results of the Optimune trial: A randomized controlled trial evaluating a novel Internet intervention for breast cancer survivors  doi: 10.1371/journal.pone.0251276 |
| Kim et al. (2025) | Evaluating the Effectiveness of a Mobile App for Breast Cancer Self-Management on Self-Efficacy: Nonrandomized Intervention Trial  doi: 10.2196/63989 |
| Schover et al. (2013) | Efficacy Trial of An Internet-Based Intervention for Cancer-Related Female Sexual Dysfunction  doi: 10.6004/jnccn.2013.0162 |
| Urech et al. (2018) | Web-Based Stress Management for Newly Diagnosed Patients With Cancer (STREAM): A Randomized, Wait-List Controlled Intervention Study  doi: 10.1200/JCO.2017.74.8491 |
| van der Hout et al. (2020) | Role of eHealth application Oncokompas in supporting self-management of symptoms and health-related quality of life in cancer survivors: a randomised, controlled trial  doi: 10.1016/S1470-2045(19)30675-8 |
| van der Hout et al. (2021) | The eHealth self-management application 'Oncokompas' that supports cancer survivors to improve health-related quality of life and reduce symptoms: which groups benefit most?  doi: 10.1080/0284186X.2020.1851764 |
| Weng et al. (2024) | Randomized controlled trial of an app for cancer pain management  doi: 10.1007/s00520-024-08442-1 |
| **Information is missing (n = 7)** | |
| Gao et al. (2022) | Effects of Tai Chi App and Facebook health education programs on breast cancer survivors? stress and quality of life in the Era of pandemic  doi: 10.1016/j.ctcp.2022.101621 |
| Heinrich et al. (2024) | Effects of mobile application‐based cognitive behavioral therapy on psychological outcomes in women treated for breast cancer: A randomized controlled pilot trial in Germany  doi: 10.1002/pon.70003. |
| Jiang et al. (2024) | Effects of the “AI-TA” mobile app with intelligent design on psychological and related symptoms of young survivors of breast cancer: Randomized controlled trial  doi: 10.2196/50783 |
| Martínez-Miranda et al. (2024) | Effectiveness of an interactive online group intervention based on pain neuroscience education and graded exposure to movement in breast cancer survivors with chronic pain: a randomised controlled trial  doi: 10.1007/s00520-024-08887-4 |
| Phillips et al. (2024) | Feasibility and preliminary effects of the Fit2ThriveMB pilot physical activity promotion intervention on physical activity and patient reported outcomes in individuals with metastatic breast cancer  doi: 10.1007/s10549-024-07432-5 |
| Owen et al. (2005) | Randomized pilot of a self-guided Internet coping group for women with early-stage breast cancer  doi: 10.1207/s15324796abm3001_7 |
| Wolff et al. (2023) | App-based lifestyle coaching (PINK!) accompanying breast cancer patients and survivors to reduce psychological distress and fatigue and improve physical activity - A feasibility pilot study  doi: 10.1159/000531495 |
